# Genetic Profiling and Comparison of Human and Animal Methicillin-Resistant *Staphylococcus aureus* (MRSA) Isolates from Serbia

**DOI:** 10.3390/antibiotics8010026

**Published:** 2019-03-16

**Authors:** Jelena Asanin, Dusan Misic, Ksenija Aksentijevic, Zoran Tambur, Bojan Rakonjac, Ivana Kovacevic, Joachim Spergser, Igor Loncaric

**Affiliations:** 1 Innovation Center of Faculty of Technology and Metallurgy, University of Belgrade, 11000 Beograd, Serbia; jelenaasanin@gmail.com; 2 Faculty of Veterinary Medicine, University of Belgrade, 11000 Beograd, Serbia; dusan@vet.bg.ac.rs (D.M.); pojavica@gmail.com (K.A.); 3 Institute of Hygiene Military Medical Academy, Belgrade, Serbia and Faculty of Stomatology in Pancevo, 11000 Beograd, Serbia; tambur.zoran@gmail.com; 4 Institute of Microbiology Military Medical Academy, 11000 Beograd, Serbia; bonatejo@gmail.com; 5 Institute of Hygiene Military Medical Academy, 11000 Beograd, Serbia; ivanchichica@gmail.com; 6 Institute of Microbiology, University of Veterinary Medicine, 1210 Wien, Austria; joachim.spergser@vetmeduni.ac.at

**Keywords:** MRSA, humans, animals, antibiotic resistance, SCC*mec* typing, *dru* typing, *spa* typing, MLVA, MLST

## Abstract

The aim of this study was to characterize a collection of methicillin-resistant *Staphylococcus aureus* (MRSA) isolates of human and animal origin from Serbia. In total, 36 MRSA isolates—30 obtained from humans and six from companion animals—were investigated by PCR for the presence of antibiotic and biocide resistance determinants and virulence genes (PVL—Panton–Valentine leukocidin, ETs—exfoliative toxins, TSST—toxic shock syndrome toxin, SEs—staphylococcal enterotoxins, and MSCRAMMs—microbial surface components recognizing adhesive matrix molecules and biofilm). Isolates were analyzed by staphylococcal cassette chromosome *mec* (SCC*mec*), *spa*, and *dru* typing, as well as by multiple locus variable number of tandem repeat analyses (MLVA), multilocus sequence typing (MLST), and subsequently, eBURST. The majority of human MRSA isolates were resistant to gentamicin, erythromycin, clindamycin, and ciprofloxacin. Different antibiotic resistance genes were detected: *aac-aphD*, *ant(6′)-Ia*, *erm*(A), *erm*(B), *erm*(C), *tet*(K), *tet*(M), *fexA*, and *cat*_pC221_. All isolates were susceptible to teicoplanin and linezolid. SCC*mec* type III was prevalent in human isolates, while SCC*mec* elements in animals were mostly nontypeable. t037 was the predominant *spa* type in human and t242 in animal MRSA isolates. The prevalent *dru* type was dt11c in human and dt10a in animal MRSA isolates. MRSA isolates exhibited 27 different MLVA types. ST239 was predominant in human, while ST5 was prevalent in canine MRSA isolates. PVL was found in two, while *tsst-1* was detected in three human isolates. Human-associated clones belonging to ST5, ST45, and ST239 MRSA clones were discovered in companion animals, which suggests anthropozoonotic transmission.

## 1. Introduction

Methicillin-resistant *Staphylococcus aureus* (MRSA) is a well-known and widespread pathogen that has the ability to cause a wide spectrum of clinical diseases in humans and various animal species [1]. MRSA strains carry a diverse and transmissible genetic element designated as staphylococcal cassette chromosome *mec* (SCC*mec*) [2]. To date, thirteen major types of SCC*mec* have been identified, as well as a number of deletion variants, composite, and irregular elements [3,4]. In MRSA isolates of human and animal origin, *mecA* and *mecC* genes have been detected [5]. MRSA strains are notorious for being multidrug-resistant [6]. In addition to *mec* genes, MRSA strains possess many different antibiotic, heavy metal, and disinfectant resistance genes, as well as virulence determinants that could be located on SCC*mec* and SCC elements, but also on the other mobile genetic elements (MGEs) [7]. 

MRSA is one of the leading causes of hospital-acquired infections. Healthcare-associated MRSA (HA-MRSA) infections represent a significant burden due to increased morbidity that leads to extended hospital stays and extra hospitalization costs but also cause higher mortality than methicillin-susceptible *S. aureus* (MSSA) [6]. At first, HA-MRSA clones were limited to hospitals and later, multidrug-resistant HA-MRSA strains moved from hospital settings to the community [6]. By contrast, community-associated *S. aureus* (CA-MRSA) emerged in the community, among healthy young individuals with no prior connections to the healthcare system. CA-MRSA strains often carry genes encoding Panton–Valentin leukocidin (PVL), which is responsible for severe infections of skin and soft tissue and necrotizing pneumonia [8]. CA-MRSA strains have become a serious public health problem, as these virulent strains rapidly spread in the general population and close social groups, causing outbreaks [8].

At present, CA-MRSA strains have shifted from the community to hospitals, where these once generally susceptible strains have acquired additional antibiotic resistance determinants [9,10].

*S. aureus* is capable of colonizing skin and mucous membranes of healthy dogs and cats, but the frequency of *S. aureus* isolation is low: generally lower than 10% [11]. Prevalence data on MRSA presence in healthy dogs and cats are variable, but most studies have reported rates of 0–6% [11].

The information on MRSA from Serbia is scarce, mainly reported on the presence and characterization of MRSA isolates from humans and food animals [12,13,14,15,16,17,18], but to date, no study on MRSA presence in companion animals has been published. 

The high proximity of companion animals to humans brings benefits for both. On the other hand, this proximity could also be associated with transmission in both directions, zoo-antrophogenic and vice versa, of antibiotic-resistant bacteria such as MRSA. Thus, due to the threat posed on animal and human health by the emergence of MRSA in humans in Serbia as well as the total scarcity of published data on MRSA from companion animals, the aim of the present study was to characterize and compare MRSA isolates originating from unrelated humans and companion animals during a certain point of time in order to determine any similarity and differences between human and animal MRSA isolates from Serbia.

## 2. Results

### 2.1. Antibiotic Susceptibility Testing 

All tested MRSA isolates originating from humans (*n* = 30) were susceptible to teicoplanin and linezolid, while 25 isolates were resistant to gentamicin and one to amikacin, and the presence of corresponding gene *aac*-*aphD* was revealed in 26 isolates, while the *ant(6′)-Ia* gene was found in 25 isolates; 22 isolates were resistant to erythromycin and clindamycin (two isolates showed inducible clindamycin resistance), which was reflected in that carrying *erm*(A) (7), *erm*(B) (7), and *erm*(C) (17) genes. Six isolates were resistant to tetracycline and the following corresponding genes were detected: *tet*(K) in 4 and *tet*(M) in 21 isolates; four isolates were resistant to chloramphenicol and matching genes *fexA* and *cat*_pC221_ were found in two and four isolates, respectively (Table 1). Resistance to ciprofloxacin was detected in 23 isolates, to rifampin in two, and to trimethoprim–sulfamethoxazole in one isolate. All MRSA isolates from animals (*n* = 6) were susceptible to amikacin, trimethoprim–sulfamethoxazole, chloramphenicol, teicoplanin, and linezolid. Resistance to gentamicin was recorded in three isolates, and the corresponding *aac*-*aphD* gene was detected in four isolates. Resistance to erythromycin and clindamycin (inducible type) was found in one isolate, and the presence of the matching *erm*(C) was revealed in that isolate. Resistance to tetracycline was exhibited by one isolate and in that isolate, corresponding *tet*(K) and *tet*(M) genes were detected. Resistance to ciprofloxacin in was found in two isolates and resistance to rifampin in one isolate, while the presence of *ant(6′)-Ia* was in revealed in three isolates (Table 1).

One human and two animal MRSA isolates were only resistant to tested β-lactam antibiotics. Multidrug resistance [19] was observed in 28 isolates originating from humans and in two isolates from animals.

The presence of following antibiotic resistance genes was not detected in any of the tested MRSA isolates: *dfrA*, *dfrD*, *dfrG*, *dfrK*; *msrA*; *tet*(L), *tet*(O); *str*; *vat*(A), *vat*(B), *vat*(C); *cfr*; *cat*_pC194_, and *cat*_pC223_ (Table 1).

### 2.2. Molecular Characterization

SCC*mec* typing of MRSA isolates of human origin revealed the presence of SCC*mec* type III as the most frequent (*n* = 16), including three isolates with SCC*mec* type III + SCCmercury, SCC*mec* type IV (*n* = 5), and I (*n* = 1). Eight MRSA isolates of human origin harbored nontypeable SCC*mec*. Two MRSA isolates of animal origin carried SCC*mec* type III and V, while in four MRSA isolates, nontypeable SCC*mec* elements were found (Table 1). 

Among MRSA isolates originating from humans, it was determined that the predominant *spa* type was t037 (*n* = 10), followed by t4789 (*n* = 4), t030 (*n* = 2), t041 (*n* = 2), t685 (*n* = 2), and single tones t024, t038, t039, t040, t044, t223, t487, t1288, and t4272. For one isolate, the protein A variable region could not be amplified by PCR. The predominant *spa* type among MRSA isolates originating from companion animals was t242 (*n* = 3), followed by single tones t127, t487, and t2029 (Table 1).

The majority of human MRSA isolates derived at the same time from the hospital revealed a uniform *dru* type—dt11c (*n* = 18). In addition to this predominant *dru* type among human isolates, the presence of several *dru* types was detected: dt10a (*n* = 4), dt8a (*n* = 2), and single tones dt8h, dt10q, dt7f, and dt10, while the *dru* type could not be determined for two isolates. The *dru* typing of animal MRSA isolates identified four *dru* types: dt10a (*n* = 3) and single tones dt7o, dt10g, and dt11c (Table 1).

The MRSA strains exhibited 27 different multiple locus variable number of tandem repeat analyses (MLVA) types (Table 1).

Among selected MRSAs, twelve sequence types (STs) could be detected (ST1, ST111, ST152, ST22, ST239, ST444, ST45, ST5, ST8, ST80, ST938, and the new one ST5907 with new *pta* allele number 626, with ST239 being predominant (*n* = 11) (Table 1).

The presence of three MRSA isolates with the same characteristics of the III-t037–dt11c–MLVA cluster 8-ST239 were detected in human specimens, while two MRSA isolates with the NT-t242-dt10a–MLVA cluster 19-ST5 were revealed from dog specimens (Table 1). 

The following staphylococcal enterotoxin (SE) genes were detected in MRSA isolates of human origin, *sea* in 23, *seg* in five, *sei* in eight, and *sej* in one isolate.

MRSA isolates of animal origin harbored *sea* (*n* = 1), *seg* (*n* = 4), *seh* (*n* = 1), and *sei* (*n* = 4).

Staphylococcal enterotoxin (SE) genes *seb*, *sec*, and *see*, and exfoliative toxin (ET) genes *eta* and *etb* were not detected in any of the human and animal isolates.

PVL genes were found in two and the *tsst-1* gene was found in three MRSA isolates originating from humans. None of the animal isolates harbored PVL and *tsst-1* genes (Table 1).

All examined immune evasion complex (IEC) genes were detected in MRSA isolates of human origin: *chp* in two, *sak* in all 30, *sea* in 16, *sep* in five, *scn* in all 30, and *hlb* in 29 isoaltes. The following IEC genes were identified in MRSA isolates of animal origin, *sea* in three, *chp* in four, *hlb* in five, and *sak* and *scn* in all six isolates (Table 1). All tested adhesine genes were found in both human and animal MRSA isolates, respectively: *fnbA* in 29 and in all six, *clfA* in 20 and in five, *clfB* in all 36, and *cna* in 20 and in one isolate. Gene that mediates biofilm formation *icaA* was detected in 21 human isolates and in five animal isolates. 

Metal resistance genes *cadD*, *copB*, and *czrC*, and QACs resistance gene *smr* were not detected in the examined human and animal MRSA isolates. One human MRSA isolate carried the *arsA* gene and one the *qacAB* gene.

## 3. Discussion

*S. aureus* is frequently isolated from specimens of human origin, considering the fact that 30% of the human population is colonized by *S. aureus* [20]. The MRSA finding in companion animals raises questions about its origin. Cats and dogs are in close contact with humans, as companion animals are often kept inside the house rather than outside [21]. This brings benefits for both sides but also risks due to transmission of pathogens [1,22,23]. On the other hand, although MRSA strains causing serious infections in dogs and cats are rarely isolated, they represent a clinical and therapeutic challenge [23]. However, zooantrophogenic transmission of MRSA has also been documented [24,25].

Results of susceptibility testing were in concordance with previously published results from Serbia with an exception of resistance to trimethoprim–sulfamethoxazole, which has not been observed in the past [13]. Generally, MRSA infections are caused by strains belonging to a several clonal complexes where CC5, CC22, and CC45 are prevalent lineages in hospitals, while CC1 and CC80 are often isolated in the community. CC8 and CC30 are pandemic and found in both hospitals and the community [26].

The prevalent *spa* type in human MRSA isolates was t037, which was previously reported in Australia, Africa as the most prevalent and Asia as the second most prevalent type. In Europe, this has been most the prevalent type in SCC*mec* III harboring strains [27]. In this study, this type was found in ten MRSA isolates, of which seven contained SCC*mec* III (including two isolates with SCC mercury) and three had nontypeable SCC*mec* elements. This t037-(SCC*mec*) III has different names, including the Brazilian, Hungarian, and Vienna clone [28]. Among ST239 *spa* types, the most common was t037, suggesting that this *spa* type represents the ancestral ST239 *spa* type [29]. ST239–MRSA-III is considered to be the oldest pandemic MRSA strain, and has been circulating in Eastern Europe, but nowadays, in most European countries, it has been replaced by other STs [8,30]. ST239 was observed in the recent study from Serbia, and it was concluded that 34 HA-MRSA isolates collected in the period of three years belonged to the single ’Serbian clade’ closely related to the Turkish clade [13]. One t037-SCC*mec* nontypable PVL-positive MRSA isolate belonged to ST152. This ST was previously described in patients tied with Macedonia and Kosovo [8] and in Serbia with a similar resistance pattern, different *spa* types, and SCC*mec* type V [16]. It has been hypothesized that the PVL-positive ST152 MRSA cluster in Central Europe originated from sub-Saharan Africa [31]. Further, isolate ST80–t044-IV possessed PVL genes. The presence of ST80–t044-IV, PVL-positive strains was previously described in a healthcare worker from Belgrade hospital and from an outpatient in Kragujevac, Serbia [14,16]. Additionally, this *spa* type was previously found in CA-MRSA isolates belonging to CC80 (t044, SCC*mec* IV, and dt10a) from Denmark, which were linked to Serbia [32]. The presence of two MRSA isolates t041 ST111-I and ST239-III was detected in this study, while the presence of this *spa* type and CC5–MRSA-I was observed in isolates from Serbia as well as Croatia [14,33]. In human MRSA isolates, two ST444–t030-III were found. The *spa* type t030 was previously reported in Serbia in hospitalized patients and healthcare workers [14,34].

One of our MRSA isolates was ST8–t024-IV. ST8-IV belongs to CA-MRSA, and it has been commonly found in Europe [35]. 

During the present study, one isolate was *spa* type t039 and belonged to ST239-III. MRSA carrying the same SCC*mec* element and belonged to the same *spa* type was isolated from a patient with osteomyelitis in Germany, but in that study, isolates were not analyzed by multilocus sequence typing (MLST) [36]. The two human isolates were ST938–t685, one harbored SCC*mec* IV, and another nontypeable SCC*mec*, and both were *tsst-1* positive. ST938 belongs to the CC30 clade, and t685 was recorded in Germany and the Netherlands and associated with CC30 [37,38] In MRSA isolate ST22–t223-IV, the *tsst-1* gene was found. This strain was susceptible to all antibiotics except beta-lactams, which has already been observed in MRSA belonged to the same *spa* and SCC*mec* type from Sicily, the Gaza Strip, Jordan, and Kuwait and belonged to ST22 [39,40]. 

In animal MRSA isolates, ST5–t242 was predominant. In two isolates, SCC*mec* was nontypeable, and in one, the SCC*mec* was type V. MRSA isolates belonging to *spa* type t242 and harboring SCC*mec* V were previously described in healthcare workers in Serbia (CC5–MRSA-V–t242) [14]. A multiresistant MRSA ST1 isolate originating from a cat with nontypeable SCC*mec* element belonged to t127, which was previously detected in SCC*mec* IV-harboring strains from Europe and in strains carrying SCC*mec* I and IV from America [27]. This *spa* type has been associated with ST1–MRSA–SCC*mec* Iva, which is one of the most common CA-MRSA strains in the UK, but it also was found in pigs, except that SCC*mec* was type V [41]. The *spa* type t2029 was detected in ST239–MRSA-III and was multidrug-resistant. This *spa* type was previously described in MRSA isolated from a patient in Cuba [42]. 

Direct repeat unit (*dru*) typing has never been applied in epidemiological analysis of MRSA isolates from Serbia. The dominant *dru* type among human MRSA isolates was dt11c. This type was found in 14 isolates carrying SCC*mec* III (t037, t038, t039, t040, t041, t487, and t4789) and in four isolates carrying nontypeable SCC*mec* elements (t037, t4789). Further, this *dru* type was found in an animal MRSA isolate belonging to t2029 harboring SCC*mec* III. This *dru* type was previously reported in MRSA belonging to CC398-V–t011 isolated from a slaughterhouse worker in Germany [43] but also as the most prevalent *dru* type in methicillin-resistant and multidrug-resistant strains of *Staphylococcus capitis* isolated from neonates in an intensive-care unit in France, Belgium, the United Kingdom, and Australia [44]. The second most prevalent *dru* type in human isolates was dt10a, which was the most prevalent *dru* type in animal isolates. In our study, this *dru* type was found in human MRSA isolates harboring SCC*mec* type IV (ST938–t685, ST5–t12886) and nontypeable SCC*mec* (ST152–t037, ST938–t685), and in animal MRSA isolates that carried SCC*mec* type V and nontypeable SCC*mec* (ST5–t242). The *dru* type dt10a is a widespread type that has previously been associated with diverse MRSA strains harboring different SCC*mec* elements and belonging to various clones [45]. The European CA-MRSA ST80–MRSA-IV strain appears to be conserved with respect to dt10a, and this type is the major *dru* type in Scottish EMRSA-15 and Denmark CC80–MRSA-IV strains [32,46]. Moreover, this *dru* type was found in both human and chicken isolates belonging to ST9 (SCC*mec* IV and t1430) [43]. Both *dru* types dt10a (SCC*mec* type IV) and dt11c (SCC*mec* type III) were found in MRSA strains belonging to ST239–t037 isolated from hospitalized patients in Malaysia [47]. The *dru* type dt8a was identified in two human MRSA isolates ST444–t030-III. This *dru* type, together with dt10a was previously detected among ST239–MRSA-III and ST22–MRSA-IV isolates [48]. In one human MRSA isolate, *dru* type dt8h was detected. This isolate belonging to ST111–t041 was the only one harboring SCC*mec* type I. The *dru* type dt8h was found in an MRSA strain isolated from a cat in Australia, and it belonged to ST22–MRSA-IV, t379 [49]. One of the human MRSA isolates—ST5907–t4272—exhibited *dru* type dt10q with nontypeable SCC*mec* element. This *dru* type was previously reported in ST398–MRSA-IV, t011 of bovine origin and chickens [43,50]. One of the human MRSA isolates—ST8–t024-IV—belonged to *dru* type dt7f. This *dru* type was previously described in ST239–MRSA-III, t030 isolated from a patient in Romania [51].

One animal MRSA isolate—ST45–t487—carried nontypeable SCC*mec* and exhibited *dru* type dt10g. This *dru* type was previously reported in different Scottish hospitals as well as in Romanian hospitals [46,52]. In one of the animal MRSA isolates, with ST1–t127 belonging to *dru* type dt7o, the SCC*mec* element was nontypeable. This *dru* type was previously described in CC1 and CC80–MRSA-IV–t127 isolated from patients in a Romanian hospital [51].

It was reported that every CC lineage had a typical collection of enterotoxin genes, for example, *sed-sej-ser* located on pathogenicity islands in CC45, while *sed-sej-ser* found on plasmid were usually detected in CC8. Further, prophage-borne *sea* was sporadically identified in CC8, CC30, CC45, and CC395. Common HA-MRSA and CA-MRSA lineages carry enterotoxin genes specific to a particular lineage [53]. According to previous reports, SEA has been the most prevalent of all SEs [54], which is in accordance with results of this study. In addition to *sea*, *sei*, and *seg* were detected as the second and third most prevalent SEs genes in humans, respectively, and the most prevalent in isolates from dogs. It has been suggested that human *S. aureus* isolates harbor the operon *egc* containing *seg* and *sei* [55], but both genes were found in *S. aureus* isolated from dogs in Portugal [56].

The IEC genes encode proteins which have specific interaction with human immune response: *chp* (chemotaxis inhibitory protein), *sak* (staphylokinase), particular enterotoxin genes as *sea*, *sep* and *sek*, *scn* (staphylococcal complement inhibitor), and *hlb* (haemolysin-β). These genes (human adaptation-related genes) are used as a marker of human origin [57]. The *sak* and *scn* genes were found in all 36 isolates, suggesting that all isolates could have human origin. 

## 4. Materials and Methods

### 4.1. Isolates

In total, 36 nonrepetitive MRSA isolates from humans (*n* = 30) and companion animals (*n* = 6, 5 canine, and 1 feline) were included in this study. Human isolates were obtained from different clinical specimens (sputum, wound, nose, ear, and skin swabs) of outpatients and inpatients hospitalized at the Military Medical Academy in Belgrade, Serbia during six months in 2016. In the same period, animal isolates were collected from swabs (wound, skin, eye, and ear) during routine diagnostics at the Department of Microbiology of the Faculty of Veterinary Medicine at the University of Belgrade. The study was conducted in accordance with the Declaration of Helsinki, and the research was approved by the Ethics Committee of the Military Medical Academy in Belgrade, Serbia. Examination of the animal samples was carried out as part of the routine bacteriological diagnostic activities at the Department of Microbiology at the Faculty of Veterinary Medicine, University of Belgrade. Therefore, according to the Good Scientific Practice of the Faculty of Veterinary Medicine at the University of Belgrade, these clinical examinations were not subject to the Faculty of Veterinary Medicine Ethics and Animal Welfare Commission reporting obligations.

### 4.2. Identification of Methicillin-Resistant Staphylococcus aureus

Samples were inoculated on Columbia agar with 5% sheep blood (bioMérieux, Marcy-l’ Etoile, France). Colonies resembling *Staphylococcus aureus* were subjected to further investigation. Isolates were identified according to standard bacteriological protocols and confirmed using matrix-assisted laser desorption/ionization–time of flight spectrometry (MALDI-TOF, Bruker, Germany). Cefoxitin resistance was confirmed by agar disk diffusion as well as by PCRs for *mecA* and *mecC* [58,59].

### 4.3. Antibiotic Susceptibility Testing 

Antibiotic susceptibility testing of *S. aureus* isolates was performed using agar disk diffusion on Mueller Hinton II Agar (Becton Dickinson, USA) according to Clinical and Laboratory Standards Institute (CLSI) standard [58]. The following antibiotics were tested; penicillin (10 U), cefoxitin (30 μg), erythromycin (15 μg), clindamycin (2 μg), chloramphenicol (30 μg), gentamicin (10 μg), amikacin (30 μg), trimethoprim–sulfamethoxazole (1.25/23.75 μg), tetracycline (30 μg), ciprofloxacin (5 μg), teicoplanin (30 μg), rifampin (5 μg), and linezolid (30 μg) (Becton Dickinson, USA). PCR was conducted for revealing the presence of the following antibiotic resistance genes: *erm*(A), *erm*(B), and *erm*(C) (confer resistance to macrolides, lincosamides, and streptogramin B); *msrA* (confers resistance to macrolides and streptogramin B); *vat*(A), *vat*(B), and *vat*(C) (confer resistance to streptogramin A); *cfr* (confers resistance to all phenicols, lincosamides, oxazolidinones, pleuromutilins, and streptogramin A); *fexA* (confers resistance to all phenicols); *cat*_pC194_, *cat*_pC221_, and *cat*_pC223_ (confer resistance to nonfluorinated phenicols–chloramphenicol); *aac*-*aphD* (confers resistance to aminoglycosides–gentamicin, kanamycin, tobramycin, and, when overexpressed, to amikacin); *ant(6′)-Ia* and *str* (confer resistance to aminoglycoside–streptomycin); *dfrA*, *dfrD*, *dfrG*, and *dfrK* (confer resistance to trimethoprim); *tet*(K) and *tet*(L) (confers resistance to tetracyclines except minocycline and glycylcyclines); *tet*(M) (confers resistance to tetracyclines, including minocycline but excluding glycylcyclines); and *tet*(O) [60,61,62,63,64,65,66,67,68,69]. 

### 4.4. Genotyping of MRSA

**Figure 1:**
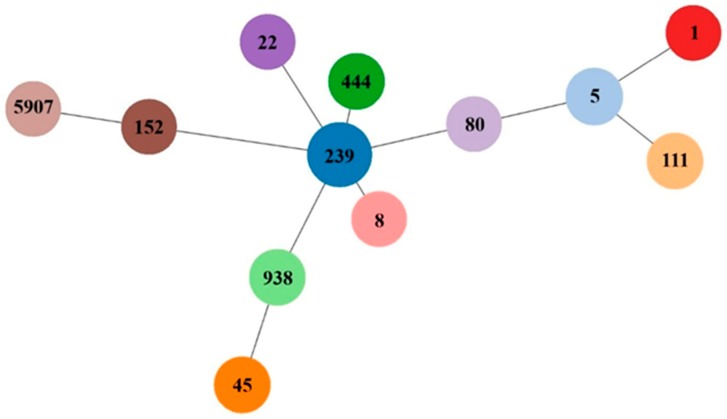
goeBURST diagram for the multilocus sequence typing (MLST) data set (STs in parenthesis) of 27 MRSA isolates. An eBURST diagram was calculated using PHYLOViZ with the goeBURST algorithm. STs were grouped according to their allelic profiles.

All strains were further genotyped by SCC*mec*, *spa* typing, and *dru* typing, by multiple locus variable number of tandem repeat analyses (MLVA) and by multilocus sequence typing (MLST) [69] of selected isolates (each representing a distinct MLVA cluster). Allele and sequence type number were assigned and used for goeBURST analysis using PHYLOViZ [70] (Figure 1).

### 4.5. Detection of Virulence and Other Determinants 

The detection of Panton–Valentine leukocidin (PVL) genes (*lukS-PV* and *lukF-PV*) as well as staphylococcal enterotoxin (SE) genes (*sea*, *seb*, *sec*, *sed*, *see*, *seg*, *seh*, *sei*, and *sej*) and the toxic shock syndrome toxin (TSST) gene (*tsst-1*) was as previously described [67]. 

Exfoliative toxin (ET) genes *eta* and *etb* were determined by the PCR protocol previously described [71].

The presence of immune evasion complex (IEC) genes—chemotaxis inhibitory protein gene (*chp*), staphylokinase gene (*sak*), staphylococcal enterotoxin A gene (*sea*), staphylococcal enterotoxin P gene (*sep*), staphylococcal complement inhibitor gene (*scn*), and haemolysin-β gene (*hlb*)—was confirmed by the PCR protocol previously described [72]. 

Microbial surface components recognizing adhesive matrix molecule (MSCRAMMs) genes: *fnbA* (fibronecting binding protein A), *clfA* and *clfB*, (clumping factors A and B), and *cna* (collagen binding protein) were targeted using the PCR protocol previously described [73].

The presence of the *icaA* gene that mediates biofilm formation was determined by the PCR protocol previously described [73]. 

Metal resistance genes *arsA* (arsenic compounds), *cadD* (cadmium), *copB* (copper), and *czrC* (zinc/cadmium) genes, as well as genes for quaternary ammonium compound (QACs) resistance (*qacAB* and *smr*) were carried out using PCR as previously described [68].

## 5. Conclusions

This study provided insight into the characteristics of MRSA strains collected from patients in one hospital in Belgrade, as well as from dogs and cat at a certain point of time. Further, this is the first report on MRSA isolates originating from dogs and cat in Serbia. The presence of ST5, ST45, and ST239 MRSA strains was revealed in specimens originating from companion animals. This discovery indicated that MRSA strains moved from hospitals not only to the community and the general population, but also to companion animals. This fact could point out that, to some degree, companion animals might be reservoirs of HA-MRSA strains.

## Figures and Tables

**Table 1:** Molecular characterization, antimicrobial resistance, and toxin profile of the methicillin-resistant *Staphylococcus aureus* isolates investigated.

									Antimicrobial Resistance		Virulence Factors		
ID	Host	Clinical Site	*spa*	*dru*	SCC*mec*	MLVA *	ST	CC	Phenotype **	Genes Detected		IEC ***	Miscellaneous Genes ****
S264	Cat	Skin swab	t127	dt7o	NT	15	ST1	CC1	β-Lactams, CIP, GEN, RIF	*mecA*, *aacA-aphD*, *ant(6’)-la*	*seg*	*sak*, *sea*, *scn*, *hlb*	*fnbA*, *clfA*, *clfB*, *icaA*
NN	Human	Wound swab	t223	nt	IV	18	ST22	CC22	β-Lactams	*mecA*	*seg*, *sei*, *tsst-1*, PVL	*chp*, *sak*, *scn*, *hlb*	*fnbA*, *clfB*
S164	Human	Sputum	t037	dt10a	NT	9	ST152	CC152	β-Lactams, CIP, GEN, TET, CHL, ERY, CLI	*mecA*,*aacA-aphD*, *ant(6′)-Ia*, *erm*(C), *tet*(K), *tet*(M), *cat*_pC221_	*sea*	*sak*, *sea*, *scn*, *hlb*	*fnbA*, *clfA*, *clfB*, *icaA*
S239	Human	Nipple discharge	t037	dt11c	III	5	ST239	CC239	β-Lactams, CIP, GEN, ERY, CLI	*mecA*, *aacA-aphD*, *ant(6′)-Ia*, *erm*(C), *tet*(M)	*sea*	*sak*, *sea*, *scn*, *hlb*	*fnbA*, *clfA*, *clfB*, *cna*, *icaA*
S241	Human	Wound swab	t037	dt11c	III + SCCmercury	6	ST239	CC239	β-Lactams, CIP, GEN, TET, ERY, CHL, CLI	*mecA*, *aacA-aphD*, *ant(6′)-Ia*, *erm*(C), *tet*(K), *tet*(M), *fexA*, *cat*_pC221_	*sea*	*sak*, *sea*, *scn*, *hlb*	*fnbA*, *clfA*, *clfB*, *cna*, *icaA*
S245	Human	Wound swab	t037	dt11c	III + SCCmercury	7	ST239	CC239	β-Lactams, CIP, GEN, AMK, TET, CHL	*mecA*, *aacA-aphD*, *ant(6′)-Ia*, *tet*(K), *tet*(M), *fexA*, *cat*_pC221_	*sea*	*sak*, *sea*, *scn*, *hlb*	*fnbA*, *clfA*, *clfB*, *cna*, *icaA*
S246	Human	Wound swab	t037	dt11c	III	8	ne	CC239	β-Lactams, CIP, GEN, ERY, CLI	*mecA*, *aacA-aphD*, *ant(6′)-Ia*, *erm*(C), *tet*(M)	*sea*	*sak*, *sea*, *scn*, *hlb*	*fnbA*, *clfA*, *clfB*, *cna*, *icaA*
S255	Human	Wound swab	t037	dt11c	III	8	ne	CC239	β-Lactams, CIP, GEN, ERY, CLI	*mecA*, *aacA-aphD*, *ant(6′)-Ia*, *erm*(C), *tet*(M)	*sea*	*sak*, *sea*, *scn*, *hlb*	*fnbA*, *clfA*, *clfB*, *cna*, *icaA*
S257	Human	Sputum	t037	dt11c	III	8	ST239	CC239	β-Lactams, CIP, GEN, ERY, CLI	*mecA*, *aacA-aphD*, *ant(6′)-Ia*, *erm*(C)	*sea*	*sak*, *sea*, *scn*, *hlb*	*fnbA*, *clfA*, *clfB*, *cna*, *icaA*
S399	Human	Wound swab	t037	dr11c	NT	10	ST239	CC239	β-Lactams, CIP, GEN	*mecA*, *aacA-aphD*, *ant(6′)-Ia*, *tet*(M)	*sea*, *sei*	*sak*, *sep*, *scn*, *hlb*	*fnbA*, *clfA*, *clfB*, *cna*, *icaA*
S401	Human	Wound swab	t037	dr11c	NT	11	ne	CC239	β-Lactams, CIP, GEN	*mecA*, *aacA-aphD*, *ant(6′)-Ia*, *tet*(M)	*sea*	*sak*, *sep*, *scn*, *hlb*	*fnbA*, *clfA*, *clfB*, *cna*, *icaA*
S473	Human	Wound swab	t037	dt11c	III	11	ne	CC239	β-Lactams, CIP, GEN	*mecA*, *aacA-aphD*, *ant(6′)-Ia*, *tet*(M)	*sea*	*sak*, *sea*, *scn*, *hlb*	*fnbA*, *clfB*, *cna*
S474	Human	Wound swab	t038	dt11c	III	11	ne	CC239	β-Lactams, CIP, GEN, ERY, CLI	*mecA*, *aacA-aphD*, *ant(6′)-Ia*, *erm*(C), *tet*(M)	*sea*, *sei*	*sak*, *sea*, *scn*, *hlb*	*fnbA*, *clfB*, *cna*
S475	Human	Wound swab	t039	dt11c	III	12	ST239	CC239	β-Lactams, CIP, GEN, ERY, CLI	*mecA*, *aacA-aphD*, *ant(6′)-Ia erm*(C), *tet*(M)	*-*	*sak*, *scn*, *hlb*	*fnbA*, *clfB*, *cna*
S476	Human	Wound swab	t040	dt11c	III	11	ne	CC239	β-Lactams, CIP, GEN	*mecA*, *aacA-aphD*, *ant(6′)-Ia*, *tet*(M)	*sea*	*sak*, *sea*, *scn*, *hlb*	*fnbA*, *clfB*, *cna*
S478	Human	Wound swab	t041	dt11c	III	11	ST239	CC239	β-Lactams, CIP, GEN, ERY, CLI	*mecA*, *aacA-aphD*, *ant(6′)-Ia*, *erm*(C), *tet*(M)	*sea*	*sak*, *sea*, *scn*, *hlb*	*fnbA*, *clfB*, *cna*
S386	Dog	Eye swab	t2029	dt11c	III	17	ST239	CC239	β-Lactams, CIP, TET, ERY, CLI (inducible)	*mecA*, *ant(6′)-Ia*, *erm*(C), *tet*(K), *tet*(M)	*sea*	*sak*, *sea*, *scn*, *hlb*	*fnbA*, *clfA*, *clfB*, *cna*, *icaA*
S400	Human	Wound swab	t4789	dr11c	NT	22	ne	CC239	β-Lactams, CIP, GEN, ERY, CLI	*mecA*, *aacA-aphD*, *ant(6′)-Ia*, *erm*(C), *tet*(M)	*sea*, *sei*	*sak*, *sep*, *scn*, *hlb*	*fnbA*, *clfA*, *clfB*, *cna*, *icaA*
S402	Human	Wound swab	t4789	dr11c	III	22	ST239	CC239	β-Lactams, CIP, GEN, ERY, CLI	*mecA*, *aacA-aphD*, *ant(6′)-Ia*, *erm*(C), *tet*(M)	*sea*	*sak*, *sep*, *scn*	*fnbA*, *clfA*, *clfB*, *cna*, *icaA*
S403	Human	Wound swab	t4789	dr11c	III	23	ST239	CC239	β-Lactams, CIP, GEN	*mecA*, *aacA-aphD*, *ant(6′)-Ia*, *tet*(M)	*sea*	*sak*, *sep*, *scn*, *hlb*	*fnbA*, *clfA*, *clfB*, *cna*, *icaA*
S480	Human	Wound swab	t4789	dt11c	NT	22	ne	CC239	β-Lactams, CIP, GEN, ERY, CLI	*mecA*, *aacA-aphD*, *ant(6′)-Ia*, *erm*(C), *tet*(M)	*sea*	*sak*, *scn*, *hlb*	*clfB*, *cna*
S479	Human	Wound swab	t487	dt11c	III	25	ST239	CC239	β-Lactams, CIP, GEN, ERY, CLI	*mecA*, *aacA-aphD*, *ant(6′)-Ia*, *erm*(C), *tet*(M)	*sea*	*sak*, *sea*, *scn*, *hlb*	*fnbA*, *clfB*, *cna*
S244a	Human	Wound swab	t685	dt10a	NT	26	ST938	CC30	β-Lactams, ERY, CLI	*mecA*, *ant(6′)-Ia*, *erm*(A), *erm*(B)	*seg*, *sei*, *tsst-1*	*sak*, *scn*, *hlb*	*fnbA*, *clfA*, *clfB*, *icaA*
S244b	Human	Wound swab	t685	dt10a	IV	27	ST938	CC30	β-Lactams, ERY, CLI	*mecA*, *erm*(A), *erm*(B)	*seg*, *sei*, *tsst-1*	*sak*, *scn*, *hlb*	*fnbA*, *clfA*, *clfB*, *icaA*
S398	Dog	Skin swab	t487	dt10g	NT	24	ST45	CC45	β-Lactams	*mecA*, *ant(6′)-Ia*	*seg*, *sei*	*chp*, *sak*, *scn*	*fnbA*, *clfA*, *clfB*, *icaA*
S395	Human	Wound swab	nt	nt	NT	1	ST111	CC5	β-Lactams, CIP, GEN, ERY, CLI, RIF	*mecA*, *aacA-aphD*, *ant(6’)-la*, *erm*(A), *erm*(B)	*sea*	*sak*, *scn*, *hlb*	*fnbA*, *clfB*, *icaA*
S258	Human	Wound swab	t041	dt8h	I	13	ST111	CC5	β-Lactams, CIP, GEN, ERY, CLI	*mecA*, *aacA-aphD*, *ant(6′)-Ia*, *erm*(A), *erm*(B), *tet*(M)	*sea*, *seg*, *sei*	*sak*, *sea*, *scn*, *hlb*	*fnbA*, *clfB*, *icaA*
S396	Human	Nose swab	t12886	dt10a	IV	16	ST5	CC5	β-Lactams, ERY, CLI (inducible)	*erm*(A), *erm*(B)	*sea*, *sed*, *seg*, *sei*	*chp*, *sak*, *scn*, *hlb*	*fnbA*, *clfA*, *clfB*, *icaA*
MRS1	Dog	Wound swab	t242	dt10a	NT	19		CC5	β-Lactams, GEN	*aacA-aphD*	*seg*, *sei*	*chp*, *sak*, *sea*, *scn*, *hlb*	*fnbA*, *clfA*, *clfB*, *icaA*
MRS2	Dog	Wound swab	t242	dt10a	NT	19	ST5	CC5	β-Lactams, GEN	*aacA-aphD*	*seg*, *sei*	*chp*, *sak*, *scn*, *hlb*	*fnbA*, *clfA*, *clfB*, *icaA*
MRS3	Dog	Ear swab	t242	dt10a	V	20	ST5	CC5	β-Lactams	*aacA-aphD*	*seg*, *sei*	*chp*, *sak*, *scn*, *hlb*	*fnbA*, *clfB*
S422	Human	Ear swab	t024	dt7f	IV	2	ST8	CC8	β-Lactams, GEN, ERY, CLI (inducible)	*aacA-aphD*, *ant(6′)-Ia*, *erm*(C)	*sej*	*sak*, *scn*, *hlb*	*fnbA*, *clfA*, *clfB*
S423	Human	Nose swab	t044	dt10	IV	14	ST80	CC80	β-Lactams, TET	*aacA-aphD*, *ant(6′)-Ia*, *tet*(K)	PVL	*sak*, *scn*, *hlb*	*fnbA*, *clfA*, *clfB*, *icaA*
S256	Human	Wound swab	t030	dt8a	III + SCCmercury	3	ST444	_	β-Lactams, CIP, GEN, TET, ERY, CLI, RIF	*aacA-aphD*, *ant(6′)-Ia*, *erm*(A), *erm*(B), *erm*(C), *tet*(M)	*sea*	*sak*, *sea*, *scn*, *hlb*	*fnbA*, *clfA*, *clfB*, *cna*, *icaA*, *qacAB*
S195	Human	Skin swab	t030	dt8a	III	4	ST444	_	β-Lactams, CIP, GEN, TET, CHL, ERY, CLI, SXT	*aacA-aphD*, *erm*(A), *erm*(B), *erm*(C), *tet*(M), *cat*_pC221_	*sea*	*sak*, *sea*, *scn*, *hlb*	*fnbA*, *clfA*, *clfB*, *cna*, *icaA*
S394	Human	Wound swab	t4272	dt10q	NT	21	ST5907	_	β-Lactams, GEN, ERY, CLI	*aacA-aphD*, *erm*(C)	*_*	*sak*, *sea*, *scn*, *hlb*	*fnbA*, *clfA*, *clfB*, *icaA*, *arsA*

* Multiple locus variable number of tandem repeat analyses (MLVA) cluster. ** CIP = ciprofloxacin; GEN = gentamicin; TET = tetracycline; CHL = chloramphenicol; ERY = erythromycin; CLI = clindamycin; RIF = rifampin; SXT = trimethoprim–sulfamethoxazole, ST = sequence type. *** Immune evasion complex (IEC) genes. **** Microbial surface components recognizing adhesive matrix molecule (MSCRAMM) genes; biofilm formation gene; metal resistance genes; quaternary ammonium compound (QAC) resistance genes.
